# Technologies, strategies and approaches for testing populations at risk of sexually transmitted infections: a systematic review protocol to inform prevention and control in EU/EEA countries

**DOI:** 10.1186/s13643-020-01303-y

**Published:** 2020-03-25

**Authors:** Suzanna C. Francis, Arun Parajuli, Otilia Mardh, Jane Falconer, Aura Andreasen, Emma Harding-Esch, on behalf of the NASSTI, Aura Andreasen, Colin S. Brown, Tania Crucitti, Kevin Dunbar, Emma Harding-Esch, Jane Falconer, Suzanna C. Francis, Jorgen S. Jensen, Steen Hoffmann, Anthony Nardone, Arun Parajuli, Keith Perry, David Mabey, Rosanna Peeling, Otilia Mardh, Silvia de San Jose, Magnus Unemo

**Affiliations:** 1 London School of Hygiene and Tropical Medicine, Keppel Street, London, WC1E 7HT UK; 2 European Centre for Disease Prevention and Control, Solna, Sweden; 3 Public Health England, London, UK

**Keywords:** Sexually transmitted infections, *Chlamydia trachomatis*, *Neisseria gonorrhoeae*, *Treponema pallidum*, *Mycoplasma genitalium*, *Trichomonas vaginalis*, Trichomoniasis, Gonorrhoea, syphilis, Diagnostic test, Coverage, Linkage to care, Access

## Abstract

**Objectives:**

This protocol outlines a systematic review methodology, aiming to assess the recent evidence-base for the impact of testing strategies and approaches on access to testing, testing coverage, and linkage to care for populations at risk for specific curable sexually transmitted infections (STIs) (chlamydia, gonorrhoea, syphilis, trichomoniasis, and *Mycoplasma genitalium* infections).

**Data sources:**

These include MEDLINE, Embase, PsycINFO, Global Health, Cochrane Database, Epistemonikos, CINAHL Plus, and Web of Science Core Collection.

**Review methods:**

Papers reporting primary data from 1 January 2012 onwards will be included. Titles, abstracts, and full texts will be reviewed for inclusion, and data will be extracted using a pre-specified and piloted data extraction form, by two independent reviewers. Experts in the field will be contacted and interviewed for further information about ongoing or unpublished studies. A narrative synthesis of the findings will be conducted.

**Discussion:**

Outcomes of this study will inform policy makers, national and international programme coordinators, public health and clinical experts, and civil society organisations involved in STI prevention and control in EU/EEA countries and elsewhere. The review will provide a direction for future researchers and programmers seeking to improve STI testing services among key populations at high risk for STIs.

**Systematic review registration:**

In accordance with guidelines outlined in the PRISMA-P methodology, this protocol was registered with the International Prospective Register of Systematic Reviews (PROSPERO) on 30 January 2019: CRD42019118261.

## Background

In 2016, there were an estimated 376.4 million new cases of the four most common curable sexually transmitted infections (STIs) worldwide: chlamydia (127.2 million cases), gonorrhoea (86.9 million cases), syphilis (6.3 million cases), and trichomoniasis (156.0 million cases) [1]. These infections have a profound impact on the health and wellbeing of people worldwide, including foetal and neonatal deaths, infertility, increased human immunodeficiency virus (HIV) transmission risk, and psychological and social consequences [2].

Compared to other regions, the prevalence and incidence of the four curable STIs in the World Health Organization (WHO) European Region were among the lowest, yet, there was substantial burden of STIs in Europe, the highest being chlamydia [3]. The 2017 European Union/European Economic Area (EU/EEA) surveillance data reflect WHO estimates and show that chlamydia, despite a large inter-country variation in testing and notification of cases, is the most frequently reported STI in Europe (409,646 cases; a notification rate of 146 per 100,000 population) followed by gonorrhoea (76,076 cases; 22 per 100,000 population) and syphilis (33,193 cases; 7 per 100,000 population) [4]. In addition, these data indicate that several sub-populations, including young people (15–24 years old), men who have sex with men (MSM), and sex workers (SW), are disproportionally diagnosed with bacterial STIs. Pregnant women from several vulnerable groups, such as migrant women and women exercising high-risk behaviours (injection drug use, sex work, etc.) were identified as at risk of adverse pregnancy outcomes due to STIs and poor access to antenatal care [5]. In addition to chlamydia, gonorrhoea, and syphilis, trichomoniasis and *Mycoplasma genitalium* are important and under-recognised causes of poor sexual health [6–8]. Poor outcomes related to curable STIs are preventable if timely and effective testing and treatment are implemented.

New testing technologies, strategies, and approaches may lead to increased coverage and enhanced delivery of public health function which would improve the prevention and control in populations most at risk for STIs. For example, rapid point-of-care tests can pave the way for decentralised STI testing including self-sampling and self-testing outside of traditional healthcare settings, including community-based organisations, pharmacies, and at home [9]. While more recent testing technologies, such as point-of-care tests, can provide faster and more flexible STI testing, they must be paired with innovative strategies and approaches for reaching populations most at risk for STIs. Indeed, innovative strategies and approaches may even utilise older technologies to increase testing access and coverage in these populations, such as a community-based approach to STI testing using a strategy of providing samples that can be self-collected and sent by post (or sample drop off locations) to an STI laboratory.

In 2012, the European Centre for Disease Prevention and Control (ECDC) published the technical report “Novel approaches to testing for sexually transmitted infections, including HIV and hepatitis B and C in Europe” [10]. This report was a comprehensive but non-systematic review of testing technologies and strategies across Europe, the USA, Canada, and Australia. It focused on the expansion of existing technology, and new technologies, such as point-of-care tests. There have been several other recent reviews on point-of-care tests, including the WHO Point-of-Care Diagnostic Landscape for STIs detailing the pipeline which is updated regularly [11–13]. The ECDC report also reviewed strategies to increase testing such as self-testing and testing through community settings, and communication of results using mHealth strategies such as SMS texting. Six years later, the aim of this review is to systematically review the literature and synthesise the results of studies implementing recent strategies and approaches in populations to improve access to testing, coverage of testing, and linkage to care. Therefore, the aim is not to review testing technologies in the pipeline (technologies in development and evaluation prior to their routine use in practice), but to gather evidence of the impact of strategies and approaches, including new testing technologies, that are currently being used. This review includes publications worldwide from 2012 onwards to capture recent strategies and approaches that could include new testing technologies reviewed earlier, and a narrowed scope to curable bacterial and parasitic STIs, as developments for testing HIV and hepatitis B and C were recent summarised in the ECDC’s public heath guidance [14]. Additionally, it requires studies to have a comparison group to be able to show a quantifiable impact of the strategies and approaches.

The primary research question of this review is “What is the impact of testing strategies and approaches that have been published since 2012, on access to testing, testing coverage and linkage to care for specific curable STIs (chlamydia, gonorrhoea, syphilis, trichomoniasis and *Mycoplasma genitalium* infection)?” The research question was formulated using the Population, Intervention, Comparison and Outcome (PICO) framework [15] (Table 1), with advice from the Novel Approaches and Strategies for STIs (NASSTI) project Advisory Committee. Secondary research questions are what testing technologies are used in these strategies and approaches to impact on access to testing, testing coverage, and linkage to care and what is the impact of testing technologies, strategies, and approaches on STI public health surveillance programmes? For example, they could lead to increased coverage and accuracy of country-level STI surveillance, earlier and more accurate diagnosis, and strengthened management and control programmes.

**Table 1:** PICO framework for identifying studies relevant to the primary research question

**P**opulation	All groups at risk for STI
**I**ntervention	Testing strategies and approaches used to increase testing access and coverage and linkage to care for *C. trachomatis*, *N. gonorrhoeae*, *T. pallidum*, *M. genitalium*, and *T. vaginalis*
**C**omparison	Any comparator
**O**utcome	Access to testing Testing coverage Linkage to care

## Methods

This systematic review protocol describes the approach to reviewing and synthesising the literature to answer the research questions. The search has been designed based on the primary research question; therefore, the data for the secondary research questions will be limited to this search strategy. In addition, experts in the field identified from the review process and other researchers who have previously worked on the topic, including the NASSTI Advisory Committee members will be contacted and interviewed for further information about ongoing or unpublished studies. This protocol is reported as per the Preferred Reporting Items for Systematic Reviews and Meta-Analyses Protocol (PRISMA-P) checklist [16] (Additional file 1). At the time of submission of this protocol for publication in April 2019, the search strategy had been finalised and was completed in November 2018, and title and abstract screening were underway.

### Search strategy

The following databases were searched: MEDLINE (OvidSP interface); Embase (OvidSP interface); PsycINFO (OvidSP interface); Global Health (OvidSP interface); Cochrane Library including Cochrane Database of Systematic Reviews, the Cochrane Central Register of Controlled Trials (CENTRAL) and Cochrane Clinical Answers (Wiley interface); Epistemonikos (Epistemonikos interface); CINAHL Plus (Ebsco interface); and the Web of Science Core Collection (Web of Science interface). The search was restricted to works published after 1st January 2012 as the previous ECDC report [10] included publications until that date. Language or geographical restrictions were not added to the search; however, studies from outside the EU/EEA or Switzerland reported in a non-English language will be excluded during screening. The terms for chlamydia, gonorrhoea, and syphilis were taken from the Cochrane STI group systematic review (2012) of topical microbicides for prevention of STIs [17]. Terms for *M. genitalium* and trichomoniasis were developed by adapting the Cochrane STI group strategy for these two infections. Two librarians were consulted to provide guidance for searching terms for “testing technologies”, “approaches”, and “strategies”. The final Medline search strategy was peer-reviewed by an ECDC librarian not associated with the project, using the Peer Review of Electronic Search Strategies (PRESS) standard [18]. The search was then adapted to meet the thesaurus terms and syntax of the other databases and published in an open access data repository (10.17037/DATA.00001047). References of included papers will be hand searched.

### Eligibility criteria and study selection

Detailed eligibility criteria are outlined in Table 2 Eligible studies must be original primary research from studies with a comparison group to show quantifiable impact (e.g. a comparison of coverage between the intervention and comparison group). Therefore, all peer-reviewed papers and conference abstracts describing studies with a comparison group were eligible, but systematic reviews were excluded. Eligible studies must be reported in English from any country or any European language from an EU/EEA country and Switzerland. The study population will be receivers of STI testing. The interventions included are testing strategies or approaches of one or more of the five pathogens: *C. trachomatis*, *N. gonorrhoeae*, *T. pallidum*, *M. genitalium*, and *T. vaginalis*. The outcomes of interest are improved access to testing, testing coverage, or linkage to care, which we have defined using WHO definitions [19] (Table 3). Due to the broad nature of the review, any comparator will be eligible for inclusion (e.g. standard practice).

**Table 2:** Eligibility criteria

**Inclusion criteria:**	
1. Papers from January 2012 to November 2018 (included in search terms)	
2. Papers investigating testing strategies or approaches of one or more of the five pathogens of interest: *C. trachomatis*, *N. gonorrhoeae*, *T. pallidum*, *M. genitalium*, and *T. vaginalis* (included in search terms)	
3. Papers reported in English from any country	
4. Papers reported in any European language from an EU/EEA country and Switzerland	
5. Papers reporting primary data	
6. Papers reporting on a testing approach or strategy for initial diagnosis of the index case, with the purpose of improving access to testing, testing coverage, or linkage to care (e.g. service evaluations)	
7. Papers reporting on genital and extra-genital (rectal and pharyngeal) infections resulting from sexual transmission in patients.	
**Exclusion criteria:**	
1. Papers reported in a non-English language outside of the EU/EEA and Switzerland (e.g. Chinese, Japanese, and Korean, or for example, a paper reported in French from Senegal)	
2. Systematic reviews, guidelines, organisational reports, abstract booklets, mathematical modelling studies	
3. Papers evaluating a testing approach or strategy not for the purpose of improving access to testing, testing coverage, or linkage to care (e.g. prevalence study only, diagnostic accuracy study, risk factor/association studies)	
4. Papers evaluating a testing approach or strategy without having a comparison group	
5. Papers evaluating a testing approach or strategy not for the purpose of initial diagnosis of the index case (e.g. studies on test of cure, partner notification, or retesting)	
6. Papers looking at testing of complications of infection (e.g. pelvic inflammatory disease (PID), tubal infertility, neurosyphilis)	
7. Papers reporting on ocular, pulmonary or other (not pharyngeal or rectal) extra-genital infection	
8. Papers reporting on animal or in vitro infections (e.g. diagnostics used in the laboratory only)	
9. Papers reporting on antimicrobial resistance (AMR) testing only

**Table 3:** WHO definitions of access, coverage and linkage to care [16]

**Access:** “Access is a broad term with varied dimensions: the comprehensive measurement of access requires a systematic assessment of the physical, economic, and socio-psychological aspects of people’s ability to make use of health services.”	
**Coverage:** “Coverage of interventions is defined as the proportion of people who receive a specific intervention or service among those who need it.”	
**Linkage to care:** e.g. proportion of infected people treated or referred for treatment, results reporting to patient, etc.	

References will be exported into EndNote software (Clarivate Analytics, Philadelphia, USA) and duplications removed. Titles, abstracts and full-text screening will be carried out independently by two reviewers (AA and AP) with the support of an eligibility criteria screening tool (Table 4). Titles and abstracts will be scanned to select full-text articles for in-depth screening. Articles will be selected for full-text review if both reviewers agree on eligibility criteria or if the abstract does not provide sufficient information to make a decision. A third reviewer (EHE or SCF) will arbitrate any discrepancies between reviewers. Inter-rater agreement will be assessed using the kappa statistic for each stage of the screening

**Table 4:** Screening tool for all stages: title, abstract, and full-text (go from steps 1 to 8)

1. Was this paper published after 1st January 2012? a. No, exclude. b. Yes or uncertain, go to step 2	
2. Does this paper involve humans? a. No (animal or in vitro; lab-only diagnostics), exclude. b. Yes or uncertain, go to step 3	
3. Is the paper in English? OR Is the paper in any European language AND from an EU/EEA country or Switzerland? a. No, exclude. b. Yes or uncertain, go to step 4	
4. Is the paper reporting primary data? a. No (systematic reviews, guidelines, organisational reports, abstract booklets, mathematical modelling studies), exclude b. Yes, go on to step 5	
5. Does this paper assess a genital and extra-genital (rectal and pharyngeal) sexually transmitted infection? a. No (other extra-genital, non-sexually transmitted infection – e.g. trachoma, congenital STIs like congenital syphilis or chlamydial conjunctivitis or pneumonia), exclude. b. Yes or uncertain, go to step 6	
6. Does this paper focus on testing strategies or approaches for infections with *C. trachomatis, N. gonorrhoeae, T. pallidum, M. genitalium* or *T. vaginalis*? a. No (e.g. HIV, HCV, BV, HSV, etc. only), exclude. b. Yes or uncertain, go to step 7	
7. Does this paper assess testing strategies or approaches to improve access to testing, testing coverage, or linkage to care? a. No, (e.g. descriptive epidemiology only, risk factor/association studies, laboratory diagnostic accuracy study, basic science leading to test development, prevention studies e.g. condom uptake, counselling, prep), exclude. b. Yes (e.g., service evaluations) or uncertain, go to step 8.	
8. Does this paper assess testing strategies or approaches for initial diagnosis of the index case? a. No (e.g. studies on test of cure, partner notification, or retesting), exclude. b. Yes or uncertain, go to step 9	
9. Does this paper assess testing strategies or approaches for complications of infection *only*? a. Yes (PID, tubal infertility, neurosyphilis, etc.), exclude. b. No or uncertain, go to step 10	
10. Is this paper reporting on AMR testing only e.g. it does not include diagnosis of the infection itself? a. Yes, exclude b. No, include.	

### Data extraction and study quality assessment tool

Data will be extracted from included studies using a pre-specified extraction form developed using a password secured online questionnaire (Online Surveys; https://www.onlinesurveys.ac.uk/). Variables to be extracted include the following: STI pathogen, study design, size of the study (e.g. number of participants enrolled), strategy or approach, impact on testing access, coverage, or linkage to care, population, setting, testing technology, reporting for surveillance, feasibility and acceptability, and gaps identified for future research. Additional data will be extracted to assess the risk of bias at the study level according to PRISMA guidelines [20]. Well-established tools will be adapted to assess the risk for bias. The Cochrane risk of bias tool (Version 1) will be used to assess randomised controlled trials [15], and the ROBINS-I tool will be used to assess risk of bias in non-randomised studies of interventions [21]. The Cochrane risk of bias tool will be adapted to introduce of an “overall bias” summary domain, similar to the ROBINS-I tool, which will allow comparison between interventional and non-interventional tools. The ROBINS-I tool will be simplified from five possible outcomes (“Low risk of bias”, “Moderate risk of bias”, “Severe risk of bias”, “Critical risk of bias”, No information) to three outcomes as suggested in the Cochrane Handbook (+ or “Low risk of bias”, − or “High risk of bias”, and ? or “Insufficient Information”)(Additional file 3).

Data will be extracted by two reviewers independently (AA and AP). Results will be compared and resolved between the two reviewers, and a third reviewer (EHE or SCF) will arbitrate any discrepancies between reviewers. The extraction form will be piloted with five papers to ensure ease-of-use, that all pertinent data items are included, and consistency between reviewers.

### Data synthesis and analysis

The final number and characteristics of studies identified for inclusion in, and exclusion from, the systematic review will be reported in a PRISMA flow diagram [20]. We will tabulate all extracted data, including participant characteristics, study designs, interventions, instruments, and study results. The data will not be synthesised quantitatively in the form of a meta-analysis due to the variety in study methodologies and outcomes anticipated in included studies. Instead, a systematic narrative synthesis will be provided with information presented in the text and tables of the included studies. For the primary research question (What is the impact of testing strategies and approaches on access to testing, testing coverage and linkage to care for curable STIs?), data will be organised by STI, population, and outcomes (access to testing, testing coverage and linkage to care). To address the secondary questions (testing technologies used; impact on STI public health surveillance programmes), we will describe the number of studies that presented data for each of the questions and present the differences between the study results in a narrative form. The narrative synthesis will explore the relationships and findings both within and between the included studies in line with the guidance from the Centre for Reviews and Dissemination [22]. Results from the risk of bias assessments will be presented in a matrix and will be used to help interpret and explain differences in results across studies. As we are not performing a quantitative synthesis, we will not use statistical methods of assessing publication bias. The strength of the body of evidence will not be assessed (e.g. GRADE) due to broad nature of the research question.

### Expert interviews and ethical considerations

After a narrative synthesis has been conducted, gaps related to the primary and secondary research questions will be identified by the study team and advisory community. Expert interviews will be conducted to further contextualise these gaps with relevant published and unpublished information provided by the expert. Any publications provide by experts will be screened for eligibility. Experts will be identified through the systematic literature review and professional networks. Each expert will be contacted by email, provided with information about the systematic review, and invited for an interview. If interested in participating, the expert will be asked for written informed consent. All consenting experts will be interviewed with a semi-structured topic guide (Additional file 2). Interviews will be held in-person or by remote telecommunications, as appropriate. Interviews will be audio-recorded with permission from the expert. For experts who do not consent to audio recording, detailed notes will be taken. The conclusions of each interview will be summarised and sent to each expert interviewee for written verification. Ethical approval has been provided by the London School of Hygiene & Tropical Medicine ethics committee (reference: 16338).

## Discussion

This systematic review will provide an evidence-base for implementing testing strategies and approaches to increase testing access, coverage, and linkage to care in populations at risk for STIs in the EU/EEA. The review will build add to the 2012 ECDC report technical report "[10], refocusing the review on specific curable STIs and strategies and approaches used to impact testing access, testing coverage and linkage to care. The latter shift is to provide a public heath perspective and inform policy makers of the approaches and strategies that can be used, even if new testing technologies cannot be feasibly implemented. Emerging STI testing technologies (i.e., diagnostics) are included as a secondary research question only (other reviews exist with this as a primary objective [11–13, 23, 24]). Additionally, this review will include studies from outside the EU/EEA as there may be strategies implemented elsewhere that may be transferable to the EU/EEA.

The strengths of this systematic review include an in-depth and well-defined search strategy for the primary research question applied to a comprehensive set of databases, no language restrictions for EU/EEA publications, and input from an advisory committee and expert interviews to address gaps identified in the search. The latter will help overcome the main limitation which is that the primary research question search will be used for the secondary research questions; therefore, increasing the risk that relevant studies will not be retrieved.

Outcomes of this study will inform policy makers, national and international programme coordinators, public health or clinical experts, and civil society organisations involved in STI prevention and control in EU/EEA countries and elsewhere. The review will provide a direction for future researchers and programmers seeking to improve STI testing services for populations at risk for STIs.

## Supplementary information

**Additional file 1.** PRISMA-P 2015 Checklist

**Additional file 2.** Expert Interview Topic Guide

**Additional file 3.** Adpated Risk of Bias tools

## Data Availability

Not applicable.

## Abbreviations

ECDC: European Centre for Disease Prevention and Control
EEA: European Economic Area
EU: European Union
HIV: Human immunodeficiency virus
MSM: Men who have sex with men
PICO: Populations, Intervention, Comparison and Outcome
PRISMA: Preferred Reporting Items for Systematic Reviews and Meta-Analyses
STI: Sexually transmitted infection
SW: Sex workers
WHO: World Health Organization
