# Supplementary material for: Technologies, strategies and approaches for testing populations at risk of sexually transmitted infections: a systematic review protocol to inform prevention and control in EU/EEA countries
Source: Syst Rev. 2020 Mar 25;9:64. doi: 10.1186/s13643-020-01303-y (PMC7098083; doi:10.1186/s13643-020-01303-y)
Supplement: Supplementary file 2 — Additional file 2. Expert Interview Topic Guide [file 13643_2020_1303_MOESM2_ESM.docx]

**Expert Interview Topic Guide**

**Time:** The interview will take approximately 30-60 minutes

**Objectives:**

1. To identify published and unpublished data on novel Sexually Transmitted Infection (STI) testing technologies, strategies, and approaches in high risk populations meeting the inclusion criteria that were not identified through the literature review.
2. To identify gaps in the current literature in the area of STI diagnostics technology, approaches, or strategies that may impact on access to testing, testing coverage, and linkage to care for populations at-risk for curable STIs; Chlamydia, Gonorrhoea, Syphilis, Trichomoniasis, and Mycoplasma.
3. To attempt to fill these gaps and allow unpublished advances in the field, following written expert verification, to be incorporated into the technical report

**Participants:** Experts in the area of novel STI testing, identified through the systematic literature review and professional networks*. Only experts who have read the preliminary report and its findings, and provided written consent, will be interviewed.*

**Materials:**

- Interviewer Notebook
- Pen
- A copy of this guide
- Preliminary Systematic Review Report
- Electronic Recorder
- Remote telecommunication (e.g. Skype)
- Information sheet and consent form

**Venue:** Participants will be interviewed in a private space to protect confidentiality, either live at LSHTM or the institution of the expert in question, or over the phone, depending on preference, location and/or availability of the interviewer.

PREPARATIONS:

- Water for key informant and Interviewer

**Introduce the interview**

“*In this systematic literature review, we aimed to follow up on the 2012 ECDC Novel STI testing report by identifying new developments in STI technologies, strategies, and approaches applicable to testing high risk groups in EU/EEA countries.*

*You were identified as an expert on this subject through…[professional network or selection of research output]. We have invited you to identify gaps in the preliminary systematic literature review report which you have been provided, and to attempt to fill these gaps with unpublished information or relevant data which may not have been identified by the systematic literature review. Furthermore, we hope to identify gaps in the literature as a whole, and fill these where possible”*

[EXPLAIN THE FOLLOWING]

- *The interview may take up to 45 minutes*
- *We would like to record the interview. All interviewees will be given a unique identifier which will be used in relation to recordings. Your name on the consent forms will be kept confidential and not attached to any electronic recordings. Data will be archived according to LSHTM procedures and will be stored on secure LSHTM and Public Health England servers. If you are comfortable with your name being acknowledged on published documents relating to this process, please initial the relevant section of the consent form.*
- *If at any point you wish to not respond or terminate the interview, please inform the interviewer.*
- *I will be happy to answer any questions you have at any point during the interview.*
- *You have been provided with an information sheet & consent form. Please sign this prior to the start of the interview. [For participants being interviewed remotely, they will be sent a copy of the consent form by secure LSHTM email. Participants will be asked to email a scanned copy of the form, with written confirmation that they have destroyed the initial copy]*

**RECORD THE TIME THE ACTIVITY STARTS**

**Topic 1:** Professional experience and work in this area.

*Please tell me about yourself and your history of working with STI Diagnostics and [Current Affiliated Organisation]*.

**Probe** [briefly, for a few minutes] along lines of questioning related to previous relevant publications, projects, clinical work related to STI diagnostics, any other information the expert would like to communicate regarding their experience in the area.

**Topic 2:** Expert assessment of the findings of the report.

1. *If your work has been included in the systematic literature review, do you have any comments on the report’s:*
2. *Summary of findings in the study/abstract?*
3. *Interpretation of its results?*
4. *Use of bias assessment tools to assess the study?*
5. *Do you have any other general comments regarding the way the report has incorporated/used your findings?*
6. *In relation to any of the other documents included through the systematic literature review, do you have any comments on the report’s:*
7. *Summary of findings in the included studies?*
8. *Interpretation of results?*
9. *Use of bias assessment tools?*
10. *Do you have any other general comments regarding the way the report has incorporated/used studies/abstracts identified through the systematic literature review?*

**Topic 3:** Identifying published and unpublished data not picked up through literature review.

1. *Through the systematic literature review … studies/abstracts were identified as meeting the specified inclusion criteria. Having examined the preliminary report, are you aware of any other published data matching the inclusions and exclusions criteria specified that were not identified in this systematic literature review? [Provide section of report with inclusion/exclusion criteria and list of included papers highlighted]*
2. *Are you aware of any unpublished data meeting the specified inclusion/exclusion criteria?*

**Topic 4:** Filling information gaps identified through literature review.

*Through the process of the systematic literature review, we identified some gaps in the literature surrounding novel STI testing technologies, strategies, and approaches. These have been summarised in the preliminary report [Provide section of report with information gaps highlighted].*

1. *We would like to explore with you the information gap areas identified to gather your views*

**Probe** each of the information gaps and encourage experts to communicate their knowledge of published or unpublished data that may answer the identified open questions.

Discuss potential approaches and areas of future research that may fill these information gaps.

1. *Can you identify any other areas of information gap; currently unanswered questions in the area of STI diagnostics technology, approaches, or strategies that may impact on access to testing, testing coverage, and linkage to care for populations at-risk to curable STIs?*

Again, **Probe** each of the information gaps and encourage experts to communicate their knowledge of published or unpublished data that may answer the identified open questions.

**End of Session**

“*This concludes the interview.* *The recording of this interview linked to an anonymous identifier number will be transcribed, and the conclusions summarized. These will be sent to you electronically for written verification, and then incorporated into the technical report with your approval. I would like to thank you for your kind contribution.”*
