# Supplementary material for: Technologies, strategies and approaches for testing populations at risk of sexually transmitted infections: a systematic review protocol to inform prevention and control in EU/EEA countries
Source: Syst Rev. 2020 Mar 25;9:64. doi: 10.1186/s13643-020-01303-y (PMC7098083; doi:10.1186/s13643-020-01303-y)
Supplement: Supplementary file 3 — Additional file 3. Adpated Risk of Bias tools [file 13643_2020_1303_MOESM3_ESM.docx]

**ADAPTED Cochrane Risk of Bias Tool, Version 1 [Randomized-Control-Trials]:**

**FOR THE FINAL QUESTION OF EACH SECTION: IF YES, CONSIDER (-) FOR SECTION, IF NO CONSIDER (+) FOR SECTION, IF INSUFFICIENT INFORMATION CONSIDER (?) FOR SECTION.**

**Bias due to issues with Random Sequence Generation**

1. a. Was a method for assignment of intervention specified? PROCEED TO B
2. Did this method include use of a random number table, computerised random number generator, or minimisation, or any other method to generate randomly assigned equal numbers? PROCEED TO 2.
3. If the design involved intentionally unequal numbers, was the randomisation ratio reported? PROCEED TO 3.
4. Is it possible the method for assignment of intervention may have led to selection bias?

**Bias due to issues with Allocation Concealment**

1. a. Was a method for concealment of allocation specified? IF NO, PROCEED TO 2. IF YES, PROCEED TO B

b. Did this method include use of an external/”third-party”/centralised technique such as a pharmacy or central telephone assignment, or automated assignment, or an internal concealed technique such as drug containers [sequentially numbered, identical], or envelopes [sequentially numbered, opaque, sealed, opened after participant details written on envelope]? PROCEED TO 2

1. Is it possible method for concealment of allocation may have led to selection bias?

**Bias due to issues with Blinding of Participants, Personnel, & Outcome Assessment**

1. a. Did the methodology clearly state which participants and personnel groups (including those performing the intervention, all patient subgroups), outcome assessor group (or groups, if different assessors were used for multiple interventions), were blinded, and was the methodology used for blinding each group laid out? IF NO, PROCEED TO B, IF YES, PROCEED TO 2.

B. Where blinding was not possible for a patient subgroup or personnel group, or assessor group, was it adequately explained why this may not be appropriate/possible. IF YES, PROCEED TO 2, IF NO, PROCEED TO C.

C. Was it otherwise apparent why blinding may not be appropriate/possible? (e.g., blinding of surgeons performing interventions) IF YES, PROCEED TO 2. IF NO PROCEED TO D.

D. Where blinding was not applied, how may the “subjective/objective” nature of the outcome have affected the impact of this lack of blinding? PROCEED TO 2.

2. Is it possible these design features may have led to performance or detection bias?

**Bias due to issues with Missing Outcome Data**

1. Were any participants with missing outcome data presented in the analysis? IF YES, PROCEED TO 2. IF NO, PROCEED TO 3.
2. What percentage of data (<10%?) was missing for each outcome? PROCEED TO 3.
3. a. Was an “as treated” analysis used [including “complete/available case”?] IF YES, PROCEED TO B. IF NO, PROCEED TO 4.

b. Is it possible this may have introduced bias due to change of group or compliance/loss- to-follow-up being associated with prognosis? PROCEED TO 4.

4. Was an imputation method used to allow an intention-to-treat analysis?) (e.g., “last observation carried forward”). PROCEED TO 5

5. a. Was number of missing patients balanced between intervention groups? IF NO, PROCEED TO B, IF YES, PROCEED TO 6

b. Is it possible this may have introduced bias due to loss to follow-up being associated with prognosis? PROCEED TO D.

1. a. Were reasons for dropout/exclusion (e.g., effect, lack of effect) presented? [Was there the possibility of dropout/exclusion due to effect in one group and lack of effect in the other]. IF YES, PROCEED TO B. IF NO, PROCEED TO 7.

b. Were reasons for dropout/exclusion balanced between intervention groups? PROCEED TO 7.

7. Is it possible missing data may have led to bias?

**Bias due to issues with Selective Reporting**

1. a. Were multiple outcomes measured and recorded & reported? PROCEED TO B.

b. Could selection from multiple recorded outcomes have led to biased conclusions?

**Other Sources of Bias**

*Design Specific Biases:*

1. a. Was the design cluster randomized? IF YES, PROCEED TO B. IF NO, PROCEED TO 2.

b. Was there differential recruitment or baseline variations between clusters, loss of clusters, or inappropriate analysis used for the cluster-randomized design? PROCEED TO C.

c. How do the results of this study compare to results found in individually randomized trials?. PROCEED TO 2.

2. a. Was a “crossover” design used? IF YES, PROCEED TO B

b. Was use of a “crossover” design suitable, and analysis suitable for this design, is it possible there was a carry-over effect post cross or was only first period data collected? PROCEED TO C.

c. How do the results of this study compare to results found in parallel group trials? PROCEED TO 3.

3. Do the results of questions 1 & 2 suggest study design may have led to biased results?

*Baseline imbalance:*

1. a. Did baseline characteristics vary between intervention group, producing evidence against the null hypothesis sufficiently strong to suggest non-randomized allocation?

*Differential diagnostic activity:*

1. Is it possible that diagnosis of outcomes may have differed between intervention groups (e.g., increased assessment due to related adverse effects; diarrhoea and prostate cancer)

*Other:*

1. Was there any evidence of recruitment of additional participants following interim result?
2. Was there any evidence of post-hoc stepping up of drug doses beyond those applicable to clinical practice?
3. Was there any evidence of any interventions before randomization?
4. Was there any evidence of contamination (pooling of drugs between intervention groups, etc.)?
5. Was there any evidence of null bias (e.g., due to excessively wide inclusion criteria)
6. Was there any evidence of insensitive instrument use?
7. Was there any evidence of fraud?

**ADAPTED ROBINS-I [Non-Randomized Studies of Interventions]:**

**FOR THE FINAL QUESTION OF EACH SECTION: IF YES, CONSIDER (-) FOR SECTION, IF NO CONSIDER (+) FOR SECTION, IF INSUFFICIENT INFORMATION CONSIDER (?) FOR SECTION.**

**Bias due to confounding**

1. Is there potential for confounding of the effect of intervention in this study? IF NO OR PROBABLY NO, PROCEED TO 4. IF YES OR PROBABLY YES, PROCEED TO 2
2. Did the authors use an appropriate analysis method that controlled for all the important confounding domains? PROCEED TO 3.
3. Where the analysis split participants’ follow up time according to intervention received & discontinuations or switches were likely to be related to factors prognostic for the outcome, did the authors use an appropriate analysis method that controlled for time-varying confounding? PROCEED TO 4.

4. Were confounding domains that were controlled for measured validly and reliably by the variables available in this study? PROCEED TO 5.

5. Did the authors control for any post-intervention variables that could have been affected by the intervention? PROCEED TO 6.

6. Is it possible confounding may have led to bias?

**Bias in selection of participants into the study**

1. a. Was selection of participants into the study (or into the analysis) based on participant characteristics observed after the start of intervention? IF YES OR PROBABLY YES PROCEED TO B. IF NO OR PROBABLY NO PROCEED TO 2.

b. Were the post-intervention characteristics that influenced selection likely to be associated with intervention? IF YES OR PROBABLY YES PROCEED TO C. IF NO OR PROBABLY NO, PROCEED TO 2.

c. Were the post-intervention characteristics that influenced selection likely to be influence

by the outcome or a cause of the outcome? IF YES OR PROBABLY YES, PROCEED TO D. IF NO OR PROBABLY NO, PROCEED TO 2.

d. Were adjustment techniques used that are likely to correct for the presence of selection biases? PROCEED TO 2.

2. a. Do start of follow-up and start of intervention coincide for most participants? [was it impossible for individuals with outcome soon after intervention to be excluded; as with use of prevalent vs. incident cases]. IF YES OR PROBABLY YES, PROCEED TO 2. IF NO OR PROBABLY NO, PROCEED TO B.

b. Were adjustment techniques used that are likely to correct for the presence of selection biases? PROCEED TO 3.

3. Is it possible selection of participants may have led to bias?

**Bias in classification of interventions**

1. a. Were intervention groups clearly defined? PROCEED TO B.

b. Was the information used to define intervention groups recorded at the start of the intervention? PROCEED TO C.

c. Could classification of intervention status have been affected by knowledge of the outcome or risk of the outcome? PROCEED TO 2.

2. Is it possible classification of interventions may have led to bias?

**Bias due to** **deviations from intended interventions**

*If your aim for this study is to assess the effect of assignment to intervention, answer questions:*

1. a. Were there deviations from the intended intervention beyond what would be expected in usual practice? PROCEED TO B.

b. Were these deviations from intended intervention unbalanced between groups and likely to have affected the outcome? PROCEED TO 2.

2. Is it possible deviation from intended interventions may have led to bias?

*If your aim for this study is to assess the effect of starting and adhering to intervention, answer questions:*

1. a. Were important co-interventions balanced across intervention groups? PROCEED TO B.

b. Were interventions implemented successfully for most participants? PROCEED TO C.

c. Did study participants adhere to the assigned intervention regimen? IF YES TO A, B, & C, PROCEED TO 2. IF NO TO A, B, OR C, PROCEED TO D.

D. Was an appropriate analysis used to estimate the effect of starting and adhering to the intervention? PROCEED TO 2.

2. Is it possible deviations from intended interventions may have led to bias?

**Bias due to missing data**

1. a. Were outcome data available for all, or nearly all, participants? [90/95%], with no participants excluded due to missing data on intervention status or other variables needed for the analysis? [confounders, etc.] IF YES PROCEED TO 2. IF NO PROCEED TO B.

B. Are the proportion of participants and reasons for missing data similar across interventions? PROCEED TO E

e. Is there evidence that results were robust to the presence of missing data? PROCEED TO E.

2. Is it possible missing data may have led to bias?

**Bias in measurement of outcomes**

1. a. Could the outcome measure have been influenced by knowledge of the intervention received? PROCEED TO B.

b. Were outcome assessors aware of the intervention received by study participants? PROCEED TO C.

c. Were methods of outcome assessment comparable across intervention groups? PROCEED TO D.

d. Were any systematic errors in measurement of the outcome related to intervention received? PROCEED TO 2.

2. Considering 1, is it possible measurement of outcomes may have led to bias?

**Bias in** **selection of the reported result**

1. Is the reported effect estimate likely to be selected, on the basis of the results, from multiple outcome measurements within the outcome domain, multiple analyses of the intervention-outcome relationship, or different subgroups? PROCEED TO 2.

2. Is it possible selection of the reported result may have led to bias?

**Overall bias**

Is it possible bias through the 7 sections above may have led to bias overall?
